# Using human genetics to understand the effect of modulating targets of antihypertensive drugs in pregnancy

**DOI:** 10.64898/2026.05.12.26352361

**Published:** 2026-05-20

**Authors:** Maria Carolina Borges, Helena Urquijo, Qian Yang, Adriaan van der Graaf, Nancy McBride, Eirin Beate Haug, Ana Goncalves Soares, Gemma C Clayton, Tom A Bond, Marwa Al Arab, Julie Horn, Laurent Thomas, Laxmi Bhatta, Bjørn Olav Åsvold, Maria C Magnus, David M Evans, Christy Burden, Katherine Birchenall, Ben Brumpton, Tom R Gaunt, Emma C Hart, Zoltan Kutalik, Deborah A Lawlor

**Affiliations:** 1 MRC Integrative Epidemiology Unit at the University of Bristol, Bristol BS1 5DS, UK; 2 Population Health Sciences, Bristol Medical School, University of Bristol, Bristol BS1 5DS, UK; 3 Department of Endocrine and Metabolic Diseases, Shanghai Institute of Endocrine and Metabolic Diseases, Ruijin Hospital, Shanghai Jiao Tong University School of Medicine, Shanghai, China; 4 Shanghai National Clinical Research Center for Metabolic Diseases, Key Laboratory for Endocrine and Metabolic Diseases of the National Health Commission of the PR China, Shanghai Key Laboratory for Endocrine Tumor, Lifecycle Health Management Center, Ruijin Hospital, Shanghai Jiao Tong University School of Medicine, Shanghai, China; 5 Department of Computational Biology, University of Lausanne, Lausanne, Switzerland; 6 HUNT Center for Molecular and Clinical Epidemiology, Department of Public Health and Nursing, Norwegian University of Science and Technology, Trondheim, Norway; 7 Frazer Institute, University of Queensland, Brisbane, Australia; 8 HUNT Research Centre, Department of Public Health and Nursing, NTNU, Norwegian University of Science and Technology, Levanger 7600, Norway; 9 Department of Obstetrics and Gynecology, Levanger Hospital, Nord-Trøndelag Hospital Trust, Levanger, Norway; 10 Division of Mental Health Care, St. Olavs Hospital, Trondheim, Norway; 11 Department of Endocrinology, Clinic of Medicine, St. Olavs Hospital, Trondheim University Hospital, Trondheim, Norway; 12 Centre for Fertility and Health, Norwegian Institute of Public Health, Oslo, Norway; 13 Institute for Molecular Bioscience, University of Queensland, Brisbane, Australia; 14 Translational Health Sciences, Bristol Medical School, University of Bristol, Bristol, UK; 15 School of Medicine and Public Health, The University of Newcastle, Australia; 16 Clinic of Medicine, St. Olavs Hospital, Trondheim University Hospital, Trondheim 7030, Norway; 17 NIHR Biomedical Research Centre, University of Bristol, Bristol, UK; 18 School of Physiology, Pharmacology and Neuroscience, University of Bristol, UK; 19 Swiss Institute of Bioinformatics, Lausanne, Switzerland; 20 University Center for Primary Care and Public Health, University of Lausanne, Lausanne, Switzerland

**Keywords:** Hypertensive disorders of pregnancy, pre-eclampsia, antihypertensives, drug target, drug safety, pregnancy, Mendelian randomization

## Abstract

**Background and Aims:**

Hypertension during pregnancy is a major cause of maternal and neonatal morbidity and mortality, yet the efficacy and safety of antihypertensive treatments in this setting remain uncertain. We evaluated the effects of antihypertensive drug targets on adverse pregnancy-related outcomes using genetic variants to instrument target perturbation.

**Methods:**

We performed drug target Mendelian randomization to mimic pharmacological perturbation of targets from six commonly used antihypertensive drug classes, using data from up to 671,922 pregnant women. Genetic variants near drug target genes associated with systolic or diastolic blood pressure were selected as instruments. We estimated effects of target modulation on six primary and eight secondary pregnancy outcomes.

**Results:**

Genetically instrumented downregulation of blood pressure through β-blocker (BB) and calcium-channel blocker (CCB) targets, particularly ADRB1 and CACNB2, was associated with a reduced risk of hypertensive disorders of pregnancy, including preeclampsia. For example, CACNB2-instrumented lowering corresponded to a 7% (95% CI: 5–9%) reduction in preeclampsia risk per 1 mmHg decrease in blood pressure. For most of the other targets, estimates were directionally consistent but imprecise. Across additional outcomes, effects varied by target, with suggestive evidence for reduced risks of miscarriage, preterm birth, small-for-gestational age birth, and labour induction, although these estimates were accompanied by substantial uncertainty.

**Conclusions:**

These findings support a protective effect of BB and CCB targets on hypertensive disorders of pregnancy and highlight potential target-specific differences in safety. This work illustrates the value of Mendelian randomization in addressing clinical uncertainties where robust trial evidence is limited.

## INTRODUCTION

One in every 10 pregnant women experiences high blood pressure during pregnancy, either due to pre-existing or pregnancy-induced hypertension (1). The incidence of hypertensive disorders of pregnancy is increasing due to rising maternal age, obesity, and a growing burden of comorbidities (2). Hypertension during pregnancy is associated with a three to five-fold higher risk of pre-eclampsia, preterm birth, small-for-gestational age (SGA) babies and perinatal death, as well as a five to ten-fold higher risk of maternal death (3, 4).

Antihypertensive treatment is usually indicated for pregnant women with hypertension (≥ 140/90 mmHg) (5–7). A 2018 Cochrane systematic review of 58 randomised controlled trials (RCTs), including data from 5,909 women with mild to moderate hypertension (140-169/90-109 mmHg), found that the use of any antihypertensive drug in pregnancy halves the risk of an episode of severe hypertension. However, evidence for effects on other clinically important outcomes was unclear, largely due to the poor-to-moderate quality of included trials and limited power to assess key safety outcomes, such as pregnancy loss or fetal growth restriction (8). Following this, an open label RCT enrolling 2,408 women with mild chronic hypertension reported that receiving antihypertensive medications, as opposed to no treatment (unless severe hypertension developed), reduced risk of a composite outcome consisting of pre-eclampsia with severe features, medically indicated preterm birth, placental abruption and fetal/neonatal death (9, 10).

There is very limited evidence on the comparative benefits and safety of different antihypertensive drug classes for pregnancy-related outcomes (6). Several guidelines consider adrenergic receptor alpha-2 agonists (AdrRA) (methyldopa), β-blockers (BB) (labetolol) and calcium channel blockers (CCB) (nifedipine) safe treatment options in pregnancy (5–7). Evidence from the 2018 Cochrane systematic review indicates that BB and CCB may exhibit greater efficacy in preventing severe hypertension episodes in pregnancy compared to AdrRA (8). In contrast, a 2025 network meta-analysis of 23 RCTs (3,989 women) found no clear evidence of superiority among labetalol, methyldopa, and nifedipine in head-to-head comparisons for severe hypertension, although labetalol showed a modest advantage for preeclampsia and preterm birth outcomes (11). Other commonly employed antihypertensive agents, such as angiotensin-converting enzyme inhibitors (ACEi) and angiotensin II receptor blockers (ARB), are contraindicated during pregnancy based on safety concerns from limited evidence (5–7), including reports of increased risks of pregnancy loss and congenital anomalies from case reports and series, cohort studies, and animal models (12–16). Taken together, these inconsistencies and evidence gaps constrain evidence-based decision-making for antihypertensive therapy in pregnancy and may contribute to medication non-adherence, heightened maternal anxiety, and avoidable risks to both mother and fetus, particularly when first-line treatments fail or when contraindicated agents are used before pregnancy recognition.

Given the limited availability of large-scale, high quality RCTs testing the effects of antihypertensive treatments during pregnancy, human genetics can provide useful insights into their on-target effect on important clinical outcomes beyond hypertensive episodes, as well as into potential differences in the efficacy and adverse effects of different classes of antihypertensive drugs. Human genetic evidence supported two-thirds of Food and Drug Administration (FDA) approved drugs in 2021 (17) and offers advantages over using animal models, which might not be directly translatable to human health. Drug target Mendelian randomization harnesses the properties of human genetic variants affecting the expression or function of proteins, which constitute most drug targets, to predict the effect of pharmacologically perturbing these drug targets (18, 19).

Previous studies have used drug target Mendelian randomization to assess the efficacy and safety of antihypertensive drugs in the general population, indicating that downregulating antihypertensive drug targets resulted in the expected lower risk of cardiovascular disease for ACEi, CCB and BB targets (20–22). In contrast, applications of drug target Mendelian randomization in pregnancy remain limited, with existing studies often underpowered (23) and restricted in the range of drug targets and outcomes considered (24, 25). Available evidence suggests that genetically proxied downregulation of BB and CCB targets may reduce the risk of preeclampsia, while downregulation of the β1-adrenergic receptor (ADRB1), targeted by BB, has been associated with lower birthweight (25). However, early analyses did not account for fetal genetic effects. More recent work indicates that the link between ADRB1 and birthweight is largely explained by the correlation between maternal and fetal genotypes, underscoring the importance of accounting for offspring genotype when interpreting drug target Mendelian randomization in the context of pregnancy (23, 24).

This study aimed to improve evidence on (i) the potential effects of antihypertensive drug treatment on a range of maternal and fetal outcomes, and (ii) the comparative benefits and risks of different antihypertensive drug classes in pregnancy. To do that, we used drug target Mendelian randomization to mimic pharmacological perturbation of targets from six commonly used types of antihypertensive drugs. We included data from up to 671,922 pregnant women and explored effects on six primary outcomes (miscarriage, stillbirth, hypertensive disorders of pregnancy, pre-eclampsia, preterm birth, and SGA) and eight secondary outcomes (induction of labour, caesarean section [C-section], elective C-section, very preterm birth, low Apgar at 5 minutes, neonatal intensive care unit [NICU] admission, as well as two continuous traits: birthweight and gestational age).

## METHODS

### Study population

We used data from study participants contributing to the MR-PREG collaboration, which is focused on improving the evidence basis for prevention and treatment of adverse outcomes during pregnancy and the perinatal period (26). At the time this study was conducted, the MR-PREG collaboration included up to 671,922 women of predominantly European ancestry from five studies — i.e. Avon Longitudinal Study of Parents and Children (ALSPAC) (27, 28); Born in Bradford (BiB) (29); the Trøndelag Health Study (HUNT) (30–33); the Norwegian Mother, Father and Child Cohort Study (MoBa) (34, 35); and UK Biobank (UKB) (36) —, as well as summary data from FinnGen (release 12) (37) and previous genome-wide association study (GWAS) metanalyses for pre-eclampsia (38), preterm birth (39) and gestational age (39). Details on study-specific characteristics are provided in **Supplementary text** and **Supplementary table 1**.

### Antihypertensive drugs and corresponding targets

We selected seven classes of drugs recommended in recent guidelines (40–42) for the treatment of hypertension in non-pregnant individuals: alpha-blocker (AB), ACEi, aldosterone receptor antagonist (AIRA), ARB), BB, CCB and thiazide/thiazide-like diuretics (TZD). We also added an eighth drug class widely used for the treatment of hypertension in pregnancy: AdrRA (5, 43). We identified a commonly used drug in each class, which was then mapped to their corresponding target(s) using the DrugBank database (44) (https://go.drugbank.com/). Targets were selected if marked as interacting directly with the drug as part of its mechanism of action (Table 1 and **Supplementary table 2**).

### Study outcomes

Outcomes were selected to align, where possible, with the primary and secondary endpoints evaluated in the largest systematic review of randomized controlled trials of antihypertensive medications (8) (**Supplementary table 3**). We pre-specified six primary outcomes (miscarriage, stillbirth, any hypertensive disorder of pregnancy (HDP), pre-eclampsia, preterm birth and SGA) and six secondary outcomes (induction of labour, C-section, elective C-section, very preterm birth, low Apgar score at 5 minutes, NICU admission). In addition, we analysed two continuous traits, birthweight and gestational age, to increase statistical power and provide complementary information to related binary outcomes. Definitions of all study outcomes, alongside those used in the reference systematic review, are provided in **Supplementary Table 3**. Detailed descriptions of data sources and outcome definitions for contributing studies in the MR-PREG collaboration have been reported previously (45), and sample sizes for each analysis are summarised in Table 2.

### Data analyses

**Figure 1.**
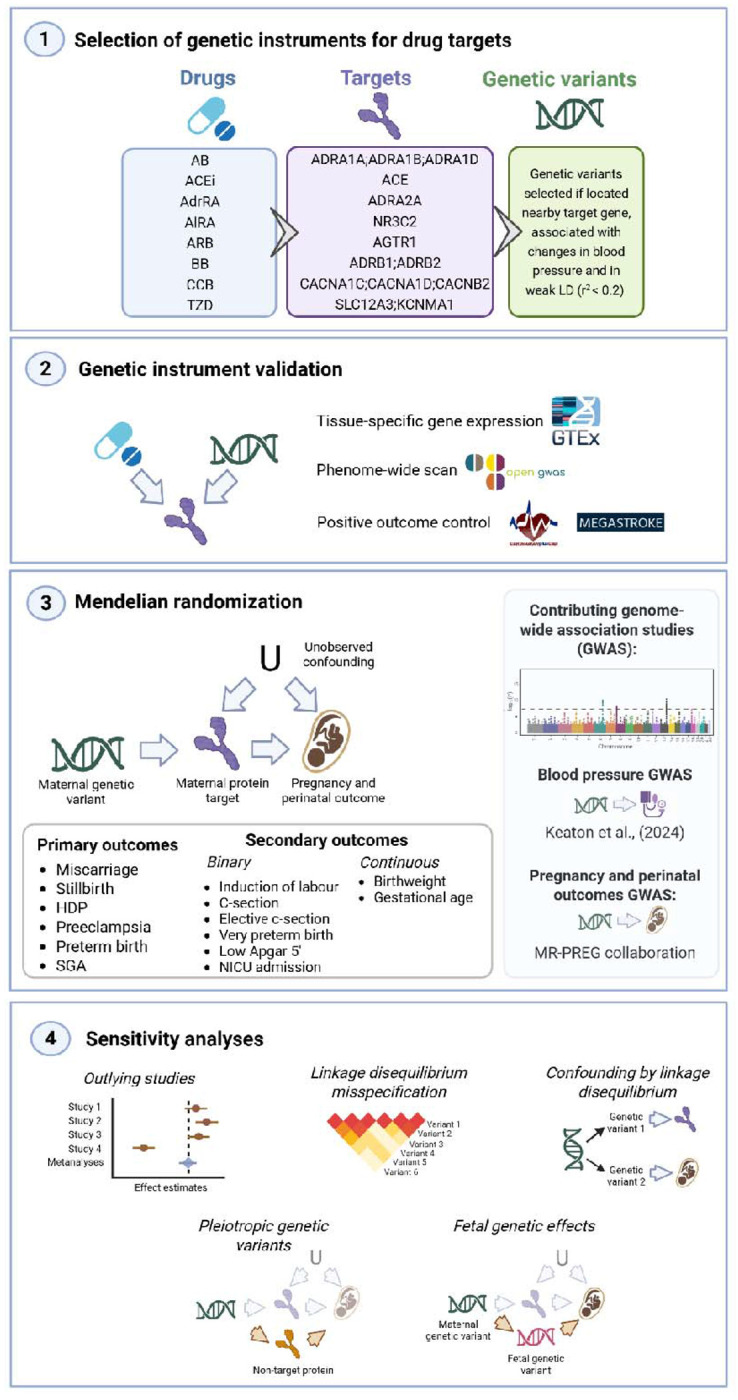
Schematic representation of the study analytical approach. Created with BioRender.com. AB: Alpha-blockers, ACEi: Angiotensin-converting enzyme inhibitors, AdrRA: Adrenergic receptor alpha-2 agonist, AlRA: Aldosterone receptor antagonist, ARB: Angiotensin II receptor blockers, BB: Beta-blockers, CCB: Calcium channel blockers, TZD: Thiazide or thiazide-like diuretics, HDP: hypertensive disorders of pregnancy, SGA, small-for-gestational age, GWAS: genome-wide association study.

Our analytical approach consists of four stages summarised in Figure 1, as described in this section. All analyses were performed using R version 4.3.1. The analytical code is available at https://github.com/mcarolborges/mr_bp-drugs_appo/. This study follows the Strengthening the Reporting of Observational Studies in Epidemiology – Mendelian Randomization (STROBE-MR) reporting guidelines (46). The study protocol was not pre-registered.

#### 1. Selection of genetic instruments for drug targets

In the Supplementary methods, we give a full description of our procedure to select genetic instruments. In brief, our main criteria were based on:

- location in the vicinity of the drug target encoding gene (aka cis-acting variants) – i.e. within a window of −/+ 500kb around the transcription start site of the target encoding gene
- potential impact on a downstream effect of the target – i.e. variants with strong evidence of association with mean changes in systolic (SBP) and/or diastolic blood pressure (DBP) at p < × 10^−8^ in a GWAS metanalysis including 1,028,980 individuals of European ancestry (47)
- limited correlation (i.e. linkage disequilibrium) with other variants selected from the same window (r^2^ < 0.2 in the main analysis and r^2^<0.01 as sensitivity analysis)

For each selected variant, we estimated the proportion of variance explained in SBP or DBP (R^2^) and the instrument strength (F-statistics).

#### 2. Characterising genetic instruments

We took a combination of approaches to explore the validity of the chosen genetic variants as suitable instruments for perturbing the drug targets. Specifically, we aimed to explore whether the selected genetic variants were likely to influence blood pressure via their impact on the respective drug targets, were specific and had the expected effect on positive control outcomes.

First, we examined whether these cis-genetic variants were associated with changes in the expression of the corresponding target genes in key tissues involved in blood pressure regulation using genetic association data from the Genotype-Tissue Expression (GTEx) project (version 8) (48). The tissues analysed included artery [aorta (N = 387), coronary (N = 213), and tibial (N = 584)], adrenal gland (N = 233), heart [atrium (N = 372), and left ventricle (N = 386)], hypothalamus (N = 170), kidney cortex (N = 73), and lung (N = 515). Where we could detect suggestive statistical evidence for an association between genetic variant and changes in gene expression in a given tissue (p < 0.05), we further investigated whether the top selected genetic variant in each locus explained more variance in the expression of the target gene than in blood pressure using the Steiger test (which would be expected if the variant primarily affects gene expression) (49) and whether the association of genetic variants with changes in gene expression and blood pressure could be explained by shared causal variants using genetic colocalization (50) (using the same approach described under ‘Confounding by LD’ below). These would be indicative that the effect of a cis-genetic variant on blood pressure is mediated by changes in the expression of the target gene. Second, we conducted a phenome-wide association scan (PheWAS) to explore the specificity of the selected genetic variants against 50,055 traits in OpenGWAS using the ieugwasr R package version (51, 52) (access: 22^nd^ July 2025). We tested whether the genetic variants had the expected effect on two positive control outcomes in non-pregnant individuals – i.e., coronary artery disease and stroke – using publicly available GWAS data from CARDIOGRAM (60,801 cases and 123,504 controls (53) and MEGASTROKE (40,585 cases and 406,111 controls) (54).

#### 3. Mendelian randomization

We used two-sample Mendelian randomization to assess the effects of modulating these drug targets on adverse pregnancy and perinatal outcomes. We present odds ratios (OR) for binary outcomes, or mean change for continuous outcomes, per 1 mmHg decrease in SBP or DBP due to genetically instrumented downregulation of the drug target gene.

For these analyses, we used genetic association data with SBP and DBP from a previous GWAS metanalyses (47) and genetic association data with pregnancy and perinatal outcomes from the MR-PREG collaboration (26). Details on quality control, GWAS analyses and meta-analyses have been published previously for these data sources are described in detail elsewhere (26, 47).

For the main analyses, we used the Wald ratio, or the generalised inverse variance weighted (gIVW) estimators if, respectively, one or multiple genetic variants were selected for a given target. The gIVW is an extension of the standard inverse variance weighted (IVW) method that accounts for linkage disequilibrium between genetic instruments (55). We used a random subsample of 10,000 unrelated UK Biobank participants of European ancestry to estimate the extent of linkage disequilibrium between variants, indicated by the r^2^, as required by gIVW. These were undertaken using the TwoSampleMR and MendelianRandomization R packages (52, 56, 57). We selected results showing some evidence of association in the main analyses (p < 0.05) for further evaluation in sensitivity analyses, and also report results corrected for multiple testing using a 5% false discovery rate (FDR) control. However, our interpretation focuses on the direction and precision of effect estimates from the main analyses, as well as their consistency across the sensitivity analyses described below, to provide a more comprehensive assessment of the evidence.

### Sensitivity analyses

As with other statistical approaches, the validity of Mendelian randomization results relies on certain assumptions, the core ones can be summarized as follows:

- Relevance: the instrument is statistically strongly associated with the exposure of interest in the relevant population (pregnant women, in this study).
- Independence: there should be no confounding between the instrument and outcome.
- Exclusion restriction criteria: any effect of the instrument on the outcome is fully mediated by the exposure.

The relevance assumption can be formally tested, whilst violations of the independence and exclusion restriction assumptions cannot be directly verified and, therefore, their plausibility was assessed through sensitivity analyses (58).

To assess instrument strength (relevance), we approximated the F-statistics as described above under ‘Selection of genetic instruments for drug targets’. Because large datasets with blood pressure measurements during pregnancy are not available, we assumed that the effects of the selected genetic instruments on blood pressure are comparable in and outside of pregnancy. Supporting this assumption, a previous Mendelian randomization study demonstrated that genetic variants strongly associated with blood pressure in the general population predicted blood pressure changes during pregnancy in the expected direction, albeit in a small but informative sample (59).

We conducted extensive sensitivity analyses to evaluate the plausibility of the exclusion restriction assumption, particularly examining potential bias due to linkage disequilibrium, pleiotropy, and fetal effects, as well as assessing the influence of outlier studies and linkage disequilibrium misspecification, as described below.

#### Outlying studies

We conducted leave-one-study-out analysis to explore potential bias due to outlying studies. First, we performed Mendelian randomization for each study separately using the same methods as in the main analyses. Second, we combined study-specific estimates using fixed effect metanalyses with gIVW after removing one study at a time.

#### Linkage disequilibrium misspecification

To assess whether results from the main analyses (gIVW) were biased by our criteria for variant selection and misspecifications of linkage disequilibrium from the reference population, we repeated analyses using IVW after using a more stringent criterion for selecting genetic variants (linkage disequilibrium clumping threshold: r^2^ < 0.01 instead of r^2^ < 0.2).

#### Confounding by linkage disequilibrium

For each drug target genomic region, we performed genetic colocalization, using the coloc R package (50), to investigate whether Mendelian randomization findings are due to shared or distinct causal genetic variants between blood pressure and pregnancy outcome. The presence of a shared variant is a necessary, although not sufficient, condition for a valid Mendelian randomization finding, whereas the presence of distinct variants would be indicative that findings are confounded by linkage disequilibrium. Coloc uses a Bayesian approach to calculate support for five causal models (H0: no association; H1: association with trait 1 only; H2: association with trait 2 only; H3: association with both traits owing to distinct causal variants; H4: association with both traits owing to a single shared causal variant). We restricted coloc analysis to a genomic region within a 500 kb window around the target gene transcription start site and assumed prior probabilities that any random single nucleotide polymorphism (SNP) in the region is associated with trait 1 (p_1_ = 1 × 10^−4^), trait 2 (p_2_ = 1 × 10^−4^) or both traits (p_12_ = 1 × 10^−5^). A posterior probability of association (PPA) for H4 ≥⍰70% was considered as evidence supporting colocalization, while PPA⍰≥⍰70% for H3 was considered as evidence against colocalization. In cases where the combined PPA for H3 and H4 was lower than 70%, which is indicative of low power for *coloc,* we performed additional sensitivity analyses by relaxing the prior for both traits (p_12_ = 1 × 10^−4^). This is justified by the fact that these analyses are based on candidate Mendelian randomization results in contrast to the hypothesis-free context in which coloc was initially proposed.

#### Pleiotropic genetic variants

Our main findings may be biased if selected genetic variants influence genes other than the intended drug target, thereby affecting pregnancy or perinatal outcomes through alternative pathways. To assess the potential impact of horizontal pleiotropy, we applied two cis-Mendelian randomization methods designed to be more robust to such effects. First, we used MR-Link2, a likelihood-based approach that jointly estimates the causal effect and the variance attributable to horizontal pleiotropy. Validation studies indicate that MR-Link2 achieves high precision, albeit with reduced recall, relative to other cis-MR methods (60). Second, we applied Generalised Summary-data-based Mendelian Randomisation (GSMR2), which detects and removes invalid instruments (i.e. defined as variants with outcome effects disproportionate to their exposure effects) while accounting for linkage disequilibrium among the remaining variants (61). Instruments were selected using p < 5 × 10^−8^ and r^2^ < 0.6. Horizontal pleiotropy was assessed using the HEIDI (Heterogeneity In Dependent Instruments) test, with a threshold of p < 0.01 to exclude potentially invalid instruments.

#### Fetal genetic effects

We accounted for a potential effect from fetal genetic variants using a weighted linear model (WLM) (62) implemented in the DONUTS R package (63). First, we used the WLM to estimate conditional genetic effects – i.e. maternal genetic effects on each outcome adjusted for offspring genotype and, for comparison, fetal genetic effects adjusted for maternal genotype. Next, we repeated the gIVW using the maternal (and fetal) conditional genetic effects on each outcome as inputs. As an additional sensitivity analyses, we further repeated this procedure using estimates of maternal genetic effects on outcomes adjusted for both offspring and paternal genotypes, to control for potential bias due to assortative mating or spurious associations between maternal and paternal variants once fetal genotype is controlled for.

## RESULTS

### Genetic instruments strength and validity

We identified genetic instruments proxying perturbation of 11 antihypertensive drug targets; no variants met inclusion criteria for ADRA2A, KCNMA1, or NR3C2. The number of instruments per target ranged from 1 to 25, with individual SNPs explaining 0.002–0.03% of blood pressure variance and indicating adequate instrument strength (F-statistics 30–408) (**Supplementary tables 4** and **5**). For most targets, blood pressure-lowering alleles of lead variants were associated with either no detectable change or reduced gene expression, most commonly in cardiovascular-relevant tissues, although opposing effects in other tissues, most commonly lung, suggest potential tissue- or cell-specific regulation (**Supplementary figure 1**). Steiger filtering supported the expected direction of effect, with variants explaining more variance in gene expression than in blood pressure (**Supplementary table 6**), and colocalisation analyses generally indicated shared causal variants, with limited inconclusive cases and one instance consistent with distinct signals [*ACE* (lung)] (**Supplementary table 7**). Phenome-wide association analyses showed that variants primarily associated with blood pressure and related traits in the anticipated direction, with weaker secondary associations across diverse phenotypes (**Supplementary figure 2** and **Supplementary table 8**). In positive control analyses, genetically proxied blood pressure reduction via most targets associated with lower risk of stroke and/or coronary artery disease, although heterogeneity was observed for specific targets, including an increased stroke risk for ADRA1B (**Supplementary table 9**). A detailed description of findings exploring instrument validity are provided in the Supplementary text.

### Mendelian randomization

#### Primary outcomes

Genetically instrumented lowering of blood pressure via BB and CCB targets, particularly ADRB1 and CACNB2, was associated with a reduced risk of HDP and preeclampsia. For illustration, ORs for preeclampsia were 0.85 (95% confidence interval (CI): 0.79, 0.92) for ADRB1 and 0.93 (95% CI: 0.91, 0.95) for CACNB2 per 1 mmHg decrease in SBP or DBP. Other BB and CCB targets showed mostly directionally consistent protective associations, although estimates were imprecise. For the remaining primary outcomes, we observed suggestive evidence of a lower risk of miscarriage (ADRB1; OR 0.97; 95% CI: 0.95, 1.00) and preterm birth (CACNA1C; OR 0.74; 95% CI: 0.60, 0.92), and a higher risk of stillbirth (CACNB2; OR 1.04; 95% CI: 1.01, 1.07); however, these did not withstand multiple testing correction (Figure 2A and **Supplementary table 10**).

For other drug classes (i.e. AB, ACEi, ARB, and TZD), we did not observe a consistent reduction in the risk of HDP and preeclampsia. Some estimates were directionally suggestive of benefit but imprecise, including HDP for ACE (OR 0.93; 95% CI: 0.85, 1.02), SLC12A3 (OR 0.89; 95% CI: 0.74; 1.08), and ADRA1A (OR 0.91; 95% CI: 0.76; 1.11), and preeclampsia for ADRA1A (OR 0.85; 95% CI: 0.69; 1.03). Most other estimates were close to the null with wide confidence intervals. One exception was a potential increase in preeclampsia risk associated with ACE downregulation (OR 1.17; 95% CI: 1.04; 1.31). We also observed suggestive evidence of reduced risk of small-for-gestational-age birth for AGTR1 (OR 0.74; 95% CI: 0.60; 0.91) and ACE (OR 0.86; 95% CI: 0.75; 0.99); however, no estimates for these drug classes passed multiple testing correction (Figure 2A and **Supplementary table 10**).

#### Secondary outcomes

For the secondary outcomes, genetically lowered blood pressure through the CCB target CACNB2 was linked to a reduced risk of labour induction (OR 0.97; 95% CI: 0.95; 0.99) and low Apgar score at 5 minutes (OR 0.87; 95% CI: 0.81; 0.93). There was also some indication that lowering blood pressure via SLC12A3 increased the risk of very preterm birth (OR 2.76; 95% CI: 1.29; 5.90) (Figure 2B). When examining continuous outcomes to maximise statistical power (i.e. birthweight and gestational age as continuous variables), we observed that genetically lowered blood pressure through ADRA1D, AGTR, ADRB2, and CACNB2 were associated with higher birthweight with estimated effects ranging from 0.01 (95% CI: 0.01; 0.02) to 0.07 (95% CI: 0.03; 0.11) SD units. Additionally, CACNB2 was associated with higher gestational age (0.01 SD; 95% CI: 0.01; 0.02), while results for the other targets were uncertain given the wide confidence intervals (**Supplementary figure 3**). Effect estimates for SLC12A3 and very preterm birth, and for ADRB2 and birthweight did not withstand multiple testing correction.

### Sensitivity analyses

Results from sensitivity analyses are presented in Figure 3, Table 3, and **Supplementary figures 4-7**. In the text, we focus on the sensitivity analysis results of drug target and (primary or secondary) outcome pairs for which there was some evidence of an effect in the main Mendelian randomization findings (p < 0.05), as summarized in Table 4.

#### Assessing the presence of influential studies

Overall, we observed consistent results in the leave-one-study-out analyses, indicating that our main findings were not driven by a specific study. In a few instances, effect estimates from the main analyses were largely or completely attenuated when removing specific studies – i.e. ADRB1 with miscarriage (UK Biobank removed); CACNA1C with HDP (FinnGen removed); CACNB2 with low Apgar at 5 minutes, NICU admission, and gestational age (MoBa removed) (**Supplementary figure 4A-C**, Table 4).

#### Examining a more stringent threshold for variant selection

Findings from Mendelian randomization analyses using a more stringent linkage disequilibrium threshold for selecting genetic variants (R^2^ < 0.01 instead of R^2^ < 0.20) were largely similar to the main analyses apart from an expected decrease in the precision of estimates (**Supplementary figure 5A-C**, Table 4).

#### Checking for confounding by linkage disequilibrium

In genetic colocalization analyses, we observed evidence supportive of a shared genetic variant for ADRA1D (birth weight), AGTR1 (birth weight), ADRB1 (HDP and pre-eclampsia), CACNA1C (preterm birth), CACNB2 (HDP and pre-eclampsia), and SLC12A3 (very preterm birth) (PPA for H4: 85-100%). In other instances, evidence for colocalization was uncertain (PPA for H1: 40-95%) rather than supportive of distinct genetic variants (PPA for H3 for other comparisons < 6%, expect for ACE and pre-eclampsia where PPA H3 = 46%) (Table 3–4, **Supplementary figures 6A-G**).

#### Accounting for fetal genetic effects

Mendelian randomization results were generally similar when accounting for potential fetal genetic effects (Table 4, **Figure 4A-B** and **Supplementary figure 7**). In some instances, adjusted estimates were inconsistent with unadjusted estimates (ACE with small-for-gestational age, AGTR1 with birthweight, CACNA1C with hypertensive disorders of pregnancy, CACNB2 with caesarean section and low Apgar score at 5 minutes), indicating that the main findings were explained by fetal, rather than maternal, effects. We could not perform adjusted analyses for miscarriage and stillbirth due to the absence of data on fetal genotype. Maternal effect estimates were similar when additionally accounting for paternal genetics (**Supplementary figure 8A-C**).

#### Exploring horizontal pleiotropy

Overall, effect estimates from our main analyses using standard Mendelian randomization methods were in the same direction when using MR-Link2 and GSMR2. In addition, there was no strong evidence of pleiotropy for most findings, except for ACE with pre-eclampsia (i.e. MR-Link2 p-value for horizontal pleiotropy < 0.05) (**Supplementary figures 9A-C**, Table 4). Estimates across methods for the positive control outcomes are presented for comparison (**Supplementary figure 10**).

## DISCUSSION

Our study utilized drug target Mendelian randomization with large-scale population data to assess the effects of perturbing eleven targets across six classes of antihypertensive drugs on various adverse pregnancy-related outcomes. Overall, our findings contribute to addressing two critical gaps in the evidence from RCTs. First, our results support the notion that downregulating blood pressure via the targets of antihypertensive drugs impacts several adverse outcomes, including preeclampsia, induction, small-for-gestational age births, and preterm deliveries. Second, our study underscores potential differences in the efficacy and safety profiles across antihypertensive drug targets.

Antihypertensive treatment for women with mild to moderate hypertension during pregnancy remains controversial. A 2018 Cochrane systematic review of RCTs found that while antihypertensive drugs halve the risk of developing severe hypertension, their impact on maternal and fetal outcomes was unclear (8). Following this, the Chronic Hypertension and Pregnancy (CHAP) trial reported that, in pregnant women with mild chronic hypertension, antihypertensive treatment (AdrRA, BB, CCB classes) lowered the risk of a composite outcome including preeclampsia, medically indicated preterm birth, placental abruption, and fetal or neonatal death compared to those randomised to no treatment (10). Our previous Mendelian randomization study supports the notion that genetically predicted lowering of systolic and diastolic blood pressure using hundreds of genetic variants across the genome reduces the risk of preterm birth, SGA birth, induction of labour, and NICU admission, but did not identify any impact on miscarriage or stillbirth risk (25). Together, these studies suggest benefits of blood pressure reduction but leave uncertainty regarding heterogeneity across drug targets and specific outcomes.

In line with the CHAP trial, our findings indicate that downregulating targets of BB and CCB is related to lower risk of preeclampsia (ADRB1, CACNB2), preterm birth (CACNA1C), and miscarriage (ADRB1), although findings for miscarriage were strongly influenced by a single study. Unexpectedly, we also identified a potential adverse association between CACNB2 downregulation, proxying CCB use, and stillbirth; however, this finding requires replication given evidence was broadly uncertain from sensitivity analyses and it did not withstand correction for multiple testing. Moreover, our results highlight potential beneficial effects of downregulating BB and CCB targets on other outcomes, including birth weight (ADRB2, CACNB2), induction of labour (CACNB2), caesarean section (CACNB2), low Apgar score at 5 minutes (CACNB2), NICU admission (CACNB2), and gestational age (CACNB2), several of which have not been adequately assessed in previous RCTs, partly due to limited statistical power. Taken together, these findings highlight the value of well-powered, well-conducted RCTs and Mendelian randomization approaches for improving our understanding of the risks and benefits associated with antihypertensive drug use during pregnancy.

Some point estimates appeared relatively large given scaling per 1 mmHg change in systolic or diastolic blood pressure. For example, the odds ratio for preeclampsia was 0.85 (95% CI: 0.79, 0.92) for genetically instrumented downregulation of blood pressure via *ADRB1*, whereas the estimate for coronary artery disease, a positive control outcome, was 0.93 (95% CI: 0.89, 0.96 per 1 mmHg), consistent with prior Mendelian randomization studies (64, 65). These magnitude of the effect estimates should be interpreted cautiously. MR estimates rely on additional assumptions to estimate the magnitude of effect, the genetic instruments may not accurately capture blood pressure changes across gestation, and uncertainty remains, as reflected in the wide confidence intervals for most estimates.

A key insight from our analyses is that direction of effects potentially vary by drug target. We observed divergent effects of blood pressure lowering across targets for preeclampsia and fetal growth outcomes (birth weight and SGA birth). For preeclampsia, downregulation of most targets was associated with the expected reduction in risk, whereas downregulation of ACE showed an apparent increase in risk. This finding should be interpreted cautiously, as it may reflect bias due to linkage disequilibrium and pleiotropy. For fetal growth outcomes, downregulation of some targets was associated with higher birth weight (ADRA1D, AGTR1, ADRB2, CACNB2) or lower risk of SGA (ACE, AGTR1), while there was weaker evidence that downregulation of ADRB1 (−0.05 SD, 95% CI: −0.04, 0; p-value = 0.051) and SLC12A3 (−0.06 SD, 95% CI: −0.12, 0; p-value = 0.053) was associated with lower birth weight. Additional analyses indicated that several of these associations were driven by fetal rather than maternal genetic effects, including ACE with SGA, and ADRB1 and AGTR1 with birth weight. Prior Mendelian randomization work has similarly reported that variants in *ADRB1* associated with lower maternal blood pressure are linked to reduced birth weight (25); our findings extend this by suggesting that the effect is primarily mediated through fetal genotype, consistent with more recent studies (23, 24). Given that several antihypertensive drugs cross the placenta and may directly affect the fetus, the contribution of fetal genetic effects to these associations is clinically relevant.

Our study has several strengths. First, we leveraged large-scale population data, which increased statistical power to assess a broader range of clinically relevant outcomes than previous RCTs and improved the generalizability of our findings. Second, the use of germline genetic variants to mimic pharmacological perturbation of antihypertensive drug targets has important advantages. This approach helps mitigate bias from confounding by indication and reverse causation, which are common in observational studies. It avoids reliance on model organisms to infer effects of target perturbation in humans. It enables the investigation of the impact of modulating targets of drugs not yet approved for use in pregnancy without exposing pregnant women to these drugs. Third, we harnessed the family-based structure of some contributing studies to disentangle maternal and fetal genetic effects. Notably, apparent adverse effects of ARBs (AGTR1) and BBs (ADRB1) on fetal growth were attributable to fetal, rather than maternal, genetic influences. This underscores the importance of accounting for fetal genetic effects in Mendelian randomization studies involving mother–offspring pairs, as well as the complexity of interpreting such findings, particularly given that most drugs cross the placenta and may exert direct effects on the fetus (66).

However, there are several considerations when translating these findings to clinical settings. First, using common genetic variants to proxy pharmacological modulation of drug targets has inherent limitations. Common genetic variants around the drug target gene are likely to reflect modest effects on the target across the life course, and therefore, may not capture non-linear effects associated with drastic downregulation of the target by pharmacological inhibition, or pinpoint critical windows of exposure (e.g. specific trimesters in pregnancy). Moreover, the mechanism of action on the target might differ between genetic variants and drugs – e.g. ACE inhibitors inhibit the activity of the enzyme, while common genetic variants are more likely to act via influencing *ACE* gene expression. Second, despite the granularity and scale of our data, including for safety outcomes such as miscarriage and stillbirth, important limitations remain. These include limited statistical power for some outcomes (e.g. very preterm birth and low Apgar score at 5 minutes), the absence of key outcomes such as congenital anomalies and developmental disorders, and the lack of large datasets with blood pressure measurements during pregnancy to assess the impact of genetic instruments on blood pressure changes during gestation. In addition, reduced power for certain targets introduces uncertainty when comparing benefit–risk profiles across drug classes and lack of offspring genotype data for non-live births precludes assessing the influence of fetal genetic effects in findings for miscarriage and stillbirth.

Our Mendelian randomization study reinforces the idea that targeting some antihypertensive drug pathways can effectively reduce the risk of hypertensive disorders of pregnancy, including pre-eclampsia. Additionally, our findings suggest the presence of target-specific mechanisms that may influence certain outcomes, especially those related to fetal growth. This study underscores the potential of Mendelian randomization as a valuable tool to provide additional evidence on the benefits and risks of antihypertensive drug use in pregnancy. This is particularly crucial given the significant knowledge gaps and the scarcity of high-quality, well-powered RCTs including pregnant people.

## Supplementary Material

Supplement 1

## Figures and Tables

**Figure 2A.**
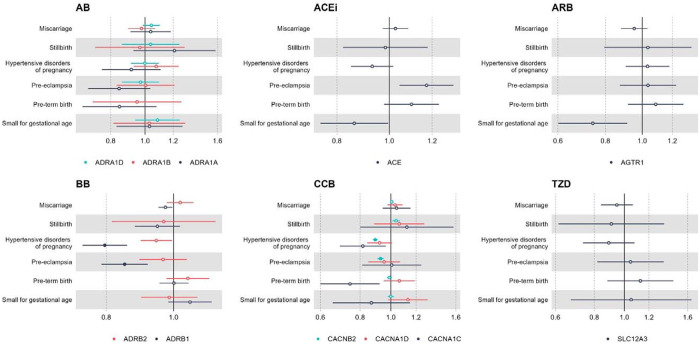
Mendelian randomization estimates for the effect of modulating hypertension drug targets on primary pregnancy-related outcomes. Results are expressed as odds ratio (OR) of outcome per 1 mmHg decrease in systolic or diastolic blood pressure (BP) due to genetically-instrumented downregulation of the drug target gene. Point estimates and 95% confidence intervals are represented by circles and bars, respectively. Filled circles indicate estimates with false discovery rate-corrected *p* values < 0.05. AB: alpha-blockers; ACE: Angiotensin-converting enzyme; ACEi: ACE inhibitors; ADRA1A: adrenergic receptor αlA; ADRA1B: adrenergic receptor αlB; ADRA1D: adrenergic receptor αlD; ADRB1: adrenergic receptor β1; ADRB2: adrenergic receptor β2; AGTR1: angiotensin II receptor type 1; ARB: angiotensin II receptor blocker; BB: beta-blockers; CACNA1C: Voltage-dependent L-type calcium channel subunit αlC; CACNA1D: Voltage-dependent L-type calcium channel subunit αlD; CACNB2: Voltage-dependent L-type calcium channel subunit β2; CCB: calcium channel blockers; SLC12A3: Solute carrier family 12 member 3; TZD: thiazide or thiazide-like diuretics.

**Figure 2B.**
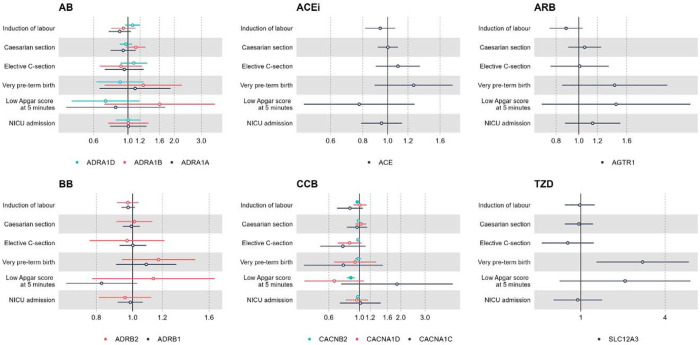
Mendelian randomization estimates for the effect of modulating hypertension drug targets on secondary binary pregnancy-related outcomes. Results are expressed as odds ratio (OR) of outcome per 1 mmHg decrease in systolic or diastolic blood pressure (BP) due to genetically-instrumented downregulation of the drug target gene. Point estimates and 95% confidence intervals are represented by circles and bars, respectively. Filled circles indicate estimates with false discovery rate-corrected *p*-values < 0.05. AB:alpha-blockers; ACE: Angiotensin-converting enzyme; ACEi: ACE inhibitors; ADRA1A: adrenergic receptor αlA; ADRA1B: adrenergic receptor αlB; ADRA1D: adrenergic receptor αlD; ADRB1: adrenergic receptor β1; ADRB2: adrenergic receptor β2; AGTR1: angiotensin II receptor type 1; ARB: angiotensin II receptor blocker; BB: beta-blockers; CACNA1C: Voltage-dependent L-type calcium channel subunit αlC; CACNA1D: Voltage-dependent L-type calcium channel subunit αlD; CACNB2: Voltage-dependent L-type calcium channel subunit β2; CCB: calcium channel blockers; SLC12A3: Solute carrier family 12 member 3; TZD: thiazide or thiazide-like diuretics.

**Figure 3A:**
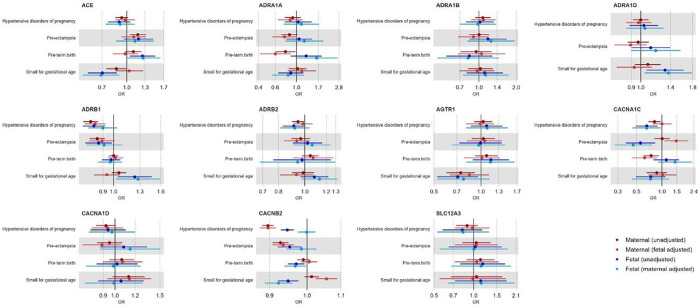
Mendelian randomization estimates for genetically-instrumented downregulation of drug targets on primary pregnancy-related outcomes based on maternal genetic effects (unadjusted and adjusted for fetal genotype) and fetal genetic effects (unadjusted and adjusted for maternal genotype). Results are expressed as odds ratio (OR) of outcome per 1 mmHg unit decrease in systolic or diastolic blood pressure (BP) due to genetically-instrumented downregulation of the drug target gene. For comparison, we present the main results for maternal effects, unadjusted and unadjusted for offspring genotype, alongside results for fetal effects, unadjusted and unadjusted for maternal genotype. Point estimates and 95% confidence intervals are represented by circles and bars, respectively. ACE: Angiotensin-converting enzyme; ADRA1A: adrenergic receptor αlA; ADRA1B: adrenergic receptor αlB; ADRA1D: adrenergic receptor αlD; ADRB1: adrenergic receptor β1; ADRB2: adrenergic receptor β2; AGTR1: angiotensin II receptor type 1; CACNA1C: Voltage-dependent L-type calcium channel subunit αlC; CACNA1D: Voltage-dependent L-type calcium channel subunit αlD; CACNB2: Voltage-dependent L-type calcium channel subunit β2; SLC12A3: Solute carrier family 12 member 3.

**Figure 3B.**
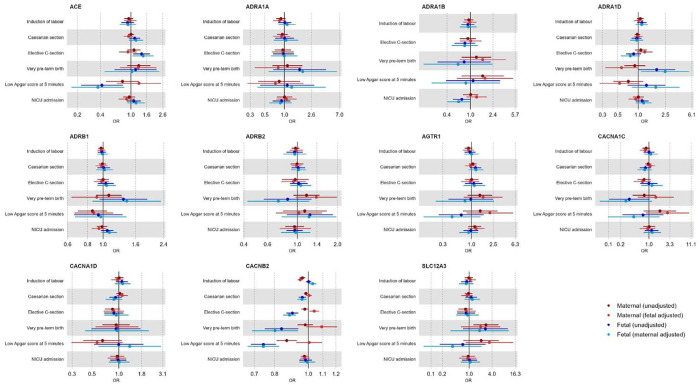
Mendelian randomization estimates for genetically-instrumented downregulation of drug targets on secondary pregnancy-related outcomes based on maternal genetic effects (unadjusted and adjusted for fetal genotype) and fetal genetic effects (unadjusted and adjusted for maternal genotype). Results are expressed as odds ratio (OR) of outcome per 1 mmHg unit decrease in systolic or diastolic blood pressure (BP) due to genetically-instrumented downregulation of the drug target gene. For comparison, we present the main results for maternal effects, unadjusted and unadjusted for offspring genotype, alongside results for fetal effects, unadjusted and unadjusted for maternal genotype. Point estimates and 95% confidence intervals are represented by circles and bars, respectively. ACE: Angiotensin-converting enzyme; ADRA1A: adrenergic receptor αlA; ADRA1B: adrenergic receptor αlB; ADRA1D: adrenergic receptor αlD; ADRB1: adrenergic receptor β1; ADRB2: adrenergic receptor β2; AGTR1: angiotensin II receptor type 1; CACNA1C: Voltage-dependent L-type calcium channel subunit αlC; CACNA1D: Voltage-dependent L-type calcium channel subunit αlD; CACNB2: Voltage-dependent L-type calcium channel subunit β2; SLC12A3: Solute carrier family 12 member 3.

**Table 1 –:** List of eligible drugs and corresponding targets

Drug class	Drug class (full name)	Drug class ATC^1^	Examples of drugs in the class	Drug target(s)^2^	UniProt ID	Recommendation in pregnancy^3^
AB	Alpha-1 adrenergic receptor antagonist (aka alpha-blocker)	C02CA	Prazosin	ADRA1A ADRA1B ADRA1D	P35348 P35368 P25100	No evidence of teratogenicity
ACEi	Angiotensin-converting enzyme inhibitors	C09AA	Enalapril	ACE	P12821	Contraindicated
AdrRA	Alpha-2 adrenergic receptor agonist	C02AB	Methyldopa	ADRA2A	P08913	Methyldopa not known to be harmful
AIRA	Aldosterone receptor antagonist	C03DA	Spironolactone	NR3C2	P08235	Use only if potential benefit outweighs risk
ARB	Angiotensin II receptor blockers	C09CA	Losartan	AGTR1	P30556	Contraindicated
BB	beta-blockers	C07A	Labetolol^4^	ADRB1 ADRB2	P08588 P07550	Labetolol not known to be harmful, except possibly in 1st trimester
CCB	Calcium channel blockers (selective)	C08CA	Nifedipine	CACNA1C CACNA1D CACNB2	Q13936 Q01668 Q08289	Use only if other treatment options are not indicated or have failed
TZD	Thiazide orthiazide-like diuretics	C03AA	Hydrochlorothiazide	SLC12A3 KCNMA1	P55017 Q12791	Contraindicated

1 ATC: Anatomical Therapeutic Chemical classification system

2 ACE: Angiotensin-converting enzyme; ADRA1A: Alpha-1A adrenergic receptor; ADRA1B: Alpha-1B adrenergic receptor; ADRA1D: Alpha-1D adrenergic receptor; ADRA2A: Alpha-2A adrenergic receptor; ADRB1: Beta-1 adrenergic receptor; ADRB2: Beta-2 adrenergic receptor; AGTR1: Type-1 angiotensin II receptor; CACNA1C: Voltage-dependent L-type calcium channel subunit alpha-1C; CACNA1D: Voltage-dependent L-type calcium channel subunit alpha-1D; CACNB2: Voltage-dependent L-type calcium channel subunit beta-2; KCNMA1: Calcium-activated potassium channel subunit alpha-1; NR3C2: Nuclear receptor subfamily 3 group C member 2; SLC12A3: Solute carrier family 12 member 3.

3 Extracted from the British National Formulary (BNF) database: https://bnf.nice.org.uk/drugs/

4 Labetolol acts as alpha- and beta-blocker, targeting ADRA1A in addition to ADRB1 and ADRB2.

**Table 2 –:** Number of cases and controls used for each outcome of interest

Outcome	Number of cases	Number of controls	Total sample size
Miscarriage	91,469	426,921	518,390
Stillbirth	6,826	227,641	234,467
Hypertensive disorders of pregnancy	32,549	509,219	541,768
Pre-eclampsia	19,408	652,584	671,992
Pre-term birth	18,225	267,497	285,722
Small for gestational age	11,874	109,340	121,214
Induction of labour	22,310	97,277	119,587
Caesarean section	33,909	200,060	233,969
Elective C-section	6,004	81,279	87,283
Very pre-term birth	1,765	100,596	102,361
Low Apgar score at 5 minutes	914	81,718	82,293
NICU admission	6,994	70,289	77,285
Birth weight			313,673
Gestational age			189,471

**Table 3.** Genetic colocalization results for blood pressure traits and adverse pregnancy outcomes according to drug target gene region

Drug class	Drug target	Trait 1	Trait 2	N SNPs	PP H0	PP H1	PP H2	PP H3	PP H4
AB	ADRA1D	DBP	Birth weight	862	0.00	0.07	0.00	0.00	0.93
ACEi	ACE	DBP	Pre-eclampsia	386	0.00	0.48	0.00	0.46	0.06
			SGA	378	0.00	0.40	0.00	0.01	0.59
ARB	AGTR1	DBP	SGA	729	0.02	0.61	0.00	0.01	0.36
			Birth weight	747	0.00	0.12	0.00	0.01	0.87
BB	ADRB1	SBP	Miscarriage	697	0.00	0.78	0.00	0.00	0.22
			HDP	670	0.00	0.00	0.00	0.01	0.99
			Pre-eclampsia	698	0.00	0.01	0.00	0.00	0.99
	ADRB2	DBP	HDP	536	0.00	0.85	0.00	0.01	0.13
			Birth weight	610	0.00	0.91	0.00	0.00	0.08
CCB	CACNA1C	DBP	HDP	603	0.00	0.44	0.00	0.03	0.52
			Preterm birth	614	0.00	0.14	0.00	0.00	0.85
	CACNB2	SBP	Stillbirth	753	0.00	0.82	0.00	0.02	0.15
			HDP	708	0.00	0.00	0.00	0.00	1.00
			Pre-eclampsia	750	0.00	0.06	0.00	0.00	0.94
			Induction of labour	720	0.00	0.87	0.00	0.06	0.07
			Caesarian section	708	0.00	0.92	0.00	0.01	0.07
			Low Apgar 5 min	747	0.00	0.73	0.00	0.04	0.22
			NICU admission	753	0.00	0.85	0.00	0.02	0.13
			Birth weight	752	0.00	0.95	0.00	0.01	0.04
			Gestational age	751	0.00	0.90	0.00	0.01	0.09
SLC12A3	SLC12A3	DBP	Very preterm birth	829	0.00	0.12	0.00	0.02	0.87

Results are expressed as posterior probabilities (PP) of association with neither trait (H0), blood pressure trait only (H1), pregnancy outcome only (H2), both traits owing to distinct genetic variants (H3), both traits owing to a shared causal variant (H4).

ACE: Angiotensin-converting enzyme; ADRA1A: adrenergic receptor α1A; ADRA1D: adrenergic receptor α1D; ADRB1: adrenergic receptor β1; ADRB2: adrenergic receptor β2; AGTR1: angiotensin II receptor type 1; ARB: angiotensin II receptor blocker; BB: beta-blockers; CACNA1C: Voltage-dependent L-type calcium channel subunit α1C; CACNB2: Voltage-dependent L-type calcium channel subunit β2; DBP: diastolic blood pressure; HDP: hypertensive disorders of pregnancy; N SNPs: number of single nucleotide polymorphisms; SBP: systolic blood pressure; SGA: small-for-gestational age; SLC12A3: Solute carrier family 12 member 3.

**Table 4.** Summary of evidence from sensitivity analyses for MR results selected for follow-up

Drug class	Drug target	exposure	Outcome	Outcome type	MR (direction)^1^	Passing FDR correction	No influential study	Robust to stringent LD	No confounding by LD	No bias by fetal genetic
AB	ADRAID	SBP	Birth weight	secondary	**+**	yes	L	*NA*	L	L
ACEi	ACE	DBP	Pre-eclampsia	primary	**+**	no	L	L	?	L
			Small for gestational age	primary	−	no	?	L	?	L
ARB	AGTR1	SBP	Small for gestational age	primary	−	no	L	*NA*	?	?
			Birth weight	secondary	**+**	yes	L	*NA*	L	L
BB	ADRB1	DBP	Miscarriage	primary	−	no	L	L	?	*NA*
			Hypertensive disorders of pregnancy	primary	−	yes	L	L	L	L
			Pre-eclampsia	primary	−	yes	L	L	L	L
	ADRB2	DBP	Hypertensive disorders of pregnancy	primary	−	no	L	L	?	?
			Birth weight	secondary	**+**	no	?	L	?	L
CCB	CACNA1C	DBP	Hypertensive disorders of pregnancy	primary	−	no	L	*NA*	?	L
			Preterm birth	primary	−	no	?	*NA*	L	L
	CACNB2	SBP	Stillbirth	primary	**+**	no	?	?	?	*NA*
			Hypertensive disorders of pregnancy	primary	−	yes	L	L	L	L
			Pre-eclampsia	primary	−	yes	L	L	L	L
			Induction of labour	secondary	−	yes	L	L	?	L
			Caesarian section	secondary	−	no	L	L	?	L
			Low Apgar score at 5 minutes	secondary	−	yes	L	?	?	L
			NICU admission	secondary	−	no	L	?	?	L
			Birth weight	secondary	**+**	yes	L	L	?	L
			Gestational age	secondary	**+**	yes	L	L	?	L
SLC12A3	SLC12A3	DBP	Very preterm birth	secondary	**+**	no	?	*NA*	L	L

**Key**	□	**?**	□
**Type of evidence**	**Not supportive**	**Uncertain**	**Supportive**
**No influential study**	Effect estimates largely attenuated to the null when removing a study from the combined estimates	Effect estimates imprecisely estimated when removing a study from the combined estimates	Effect estimates in the same direction and of similar magnitude when removing each study at a time from the combined estimates
**Robust to stringent LD**	Effect estimates attenuated to the null or in the opposite direction	Effect estimates largely attenuated to the null	Effect estimates in the same direction and comparable magnitude
**No confounding by LD**	PP H3 > 0.70	PP H4 < 0.70 and PP H3 < 0.70	PP H4 > 0.70
**No bias by fetal genetics**	Maternal effect estimates largely attenuated to the null with strong evidence of direct fetal genetic effects	Maternal effect estimates partly attenuated to the null with some evidence of direct fetal genetic effects	Maternal effect estimates in the same direction and of comparable magnitude after accounting for fetal genotype
**No evidence of pleiotropy**	Effect estimates in the opposite direction, or attenuated towards the null with p-value for bias <0.05	Effect estimates attenuated to the null with overlapping confidence intervals and p-value for bias ≥ 0.05	Effect estimates in the same direction with p-value for bias ≥ 0.05

1 Direction of MR effect estimates reflecting higher (+) or lower (−) risk of the outcome per unit decrease in blood pressure (BP) due to genetically-instrumented downregulation of the drug target gene. LD: linkage disequilibrium.

## Data Availability

Genetic association data for the exposure are publicly available on the GWAS Catalog website data repository (https://www.ebi.ac.uk/gwas) with data accession codes GCST90310294 and GCST90310295 for SBP and DBP, respectively. Genetic association data for the outcomes, generated by the MR-PREG collaboration, can only be used for research that is covered by data agreements with current contributing studies. The ALSPAC access policy that describes the proposal process in detail including any costs associated with conducting research at ALSPAC, which may be updated from time to time, and is available at: https://www.bristol.ac.uk/media-library/sites/alspac/documents/researchers/data-access/ALSPACAccessPolicy.pdf. Data is available upon request from BiB, and information is available at: https://borninbradford.nhs.uk/research/how-to-access-data/⍰ Data from MoBa are available upon application to Helsedata administered by the Norwegian Institute of Public Health (see its website https://www.fhi.no/en/ch/studies/moba/for-forskere-artikler/research-and-data-access/ for details). Data from the HUNT Study are available upon application to HUNT Research Centre, and information is available at: https://www.ntnu.edu/hunt/research Researchers can apply for access to the UK Biobank data via the Access Management System (AMS) (https://www.ukbiobank.ac.uk/enable-your-research/apply-for-access).
